# Reconstruction of Chronic Quadriceps and Achilles Tendon Ruptures Using Achilles Allografts: Clinical Findings and Review of Literature

**DOI:** 10.3390/biomedicines13040816

**Published:** 2025-03-28

**Authors:** Cătălin-Adrian Miu, Mihai Hurmuz, Luminița-Oana Miu, Daniel Ceachir, Romulus-Fabian Tatu

**Affiliations:** 1 Department XV, Discipline of Orthopedics, “Victor Babeș” University of Medicine and Pharmacy Timisoara, 300041 Timisoara, Romania; miu.catalin@umft.ro (C.-A.M.); daniel.ceachir@umft.ro (D.C.); tatu.fabian@umft.ro (R.-F.T.); 2 Orthopedics Unit, “Dr. Victor Popescu” Emergency Military Clinical Hospital, 300080 Timisoara, Romania; 3 Radiology and Medical Imaging Unit, Municipal Emergency Clinical Hospital, 300254 Timisoara, Romania; droanamiu@yahoo.com

**Keywords:** tendon allograft, chronic tendon rupture, patellar tendon, Achilles tendon, posterior cruciate ligament, surgical reconstruction

## Abstract

**Background/Objectives:** Chronic ruptures of the quadriceps and Achilles tendons present significant reconstructive challenges due to factors such as tendon retraction, scar tissue formation, and compromised tissue quality. Traditional repair methods, including V–Y tendinoplasty, autografts, and synthetic scaffolds, often prove inadequate for large or neglected defects. Achilles tendon bone–tendon allografts have emerged as a promising alternative, offering strong fixation, biological incorporation, and sufficient length for bridging extensive gaps. This study aims to document the clinical, radiographic, and MRI outcomes of two challenging cases treated with Achilles tendon bone–tendon allografts and to synthesize these findings within the context of the existing literature to evaluate the broader viability of this reconstructive approach. **Methods:** An observational analysis was conducted at the Orthopedic and Traumatology Clinic of “Victor Popescu” Military Emergency Hospital in Timișoara, encompassing two patients with chronic, iterative tendon ruptures—one quadriceps tendon rupture and one Achilles tendon rupture. Both patients had previously failed primary repairs, resulting in significant tendon retraction and tissue deficits. Reconstruction was performed using Achilles tendon bone–tendon allografts, involving specific osteotomy techniques for patellar and calcaneal fixation. Postoperative protocols included immobilization followed by structured physiotherapy. Clinical assessments and MRI evaluations were conducted at 8, 12, and 24 weeks postoperatively. Additionally, a comprehensive literature review was performed to compare our findings with existing studies on Achilles bone–tendon allograft utilization in chronic tendon reconstructions. **Results:** Both patients exhibited substantial improvements in their range of motion and reported low pain levels at the 8- and 12-week follow-ups. MRI assessments indicated well-aligned graft fibers, early bone block integration, and the absence of complications such as re-rupture or infection in the long term. Functional recovery was achieved with complete bone block union and return to normal activities by 24 weeks. The literature review corroborated these outcomes, demonstrating that Achilles tendon bone–tendon allografts provide robust fixation and facilitate biological integration, particularly in cases with large defects and poor tissue quality. Comparative studies highlighted similar functional improvements and graft stability, reinforcing the efficacy of bone–tendon allograft constructs over traditional repair methods in chronic tendon ruptures. **Conclusions:** Achilles tendon bone–tendon allografts are effective in reconstructing chronic quadriceps and Achilles tendon ruptures, offering robust fixation and facilitating biological integration. These findings, supported by the existing literature, suggest that Achilles bone–tendon allografts are a viable alternative to traditional repair strategies, especially in patients with extensive tendon defects and compromised tissue quality. Further comparative studies are warranted to establish the superiority of bone–tendon allograft constructs over conventional methods.

## 1. Introduction

Chronic tendon ruptures, whether involving the quadriceps, rotator cuff, biceps, or the Achilles tendon, are particularly difficult to manage due to tendon retraction, scar formation, and potential comorbidities such as obesity and degenerative tissue changes [1,2]. Primary tendon repair is often inadequate for large or chronically neglected defects because the tendon ends may be retracted or enveloped in scar tissue, creating a gap that cannot be closed without excessive tension or compromise to the blood supply [3,4]. In these scenarios, the objective is to restore functional continuity of the muscle–tendon unit while ensuring stable fixation that allows early mobilization and rehabilitation [5,6]. Advances in graft technology have led to the increased use of allografts, most notably Achilles tendon bone–tendon allografts, which offer several biomechanical advantages, by providing comparable biomechanical strength and elasticity to autografts while avoiding donor site morbidity and reducing surgical time [7,8,9,10]. The inclusion of a calcaneal bone block permits strong bone-to-bone healing, and the robust tendon portion is well suited to bridging larger gaps in either the quadriceps or the Achilles tendon [11,12].

Achilles bone–tendon allografts have been employed primarily in the context of Achilles tendon reconstruction, especially when the gap is extensive or when re-ruptures occur after failed primary repairs [13,14]. However, their application has extended to quadriceps tendon ruptures, most notably in cases where local tissue is insufficient or in iterative ruptures following unsuccessful initial surgeries [15]. The bone block component can be adapted to either the patella (for quadriceps reconstruction) or the calcaneus (for Achilles reconstruction), providing a stable platform for fixation [2]. As with any allograft, considerations include the risk of immunological response, disease transmission, and proper storage and sterilization [16]. Modern tissue banks and advanced processing methods have largely minimized these concerns, but surgeons must remain cognizant of potential complications [17].

Restoring function in chronic tendon ruptures requires more than just a structurally sound repair; it hinges on securing sufficient graft integrity to withstand rehabilitation demands [18,19]. Postoperative protocols generally involve a period of immobilization, followed by gradual loading exercises designed to stimulate collagen alignment and muscle reconditioning [20,21]. The introduction of an early yet protected range of motion helps prevent stiffness and excessive scar formation, both of which can impede long-term function [22,23]. In cases of chronic tendon ruptures, MRI is crucial for assessing successful graft integration by showing a reduction in signal intensity at the graft site over time, which indicates decreasing inflammation and increasing tendon maturation [24]. Successful outcomes are often correlated with the appearance of a more organized, linear collagen fiber alignment within the graft, resembling that of a normal tendon [24]. Additionally, MRI can effectively monitor the absence of fluid collections or persistent gaps at the repair site, which are signs of a positive healing response and graft functionality [25]. Given the high stakes associated with returning to normal gait patterns, sports activities, or the basic activities of daily living, the monitoring and documentation of these processes are crucial for patient safety and outcome optimization.

Beyond graft-related parameters, individual patient factors must be taken into account when selecting a reconstructive method [26]. Chronic ruptures often involve secondary changes in muscle architecture, including atrophy and fibrotic replacement, which can prolong rehabilitation [27]. In addition, metabolic factors such as diabetes or kidney disease can impair tissue healing, while obesity can contribute to elevated mechanical loads that challenge even the most robust repairs [28,29]. Surgical expertise is also pivotal: achieving adequate tension, properly positioning the bone block, and ensuring meticulous suture technique can all influence clinical success.

While case reports and smaller observational studies have been published detailing success with Achilles tendon bone–tendon allografts for both quadriceps and Achilles reconstructions, robust comparative data remain limited [30]. Comparing allografts to traditional repair strategies like V-Y tendinoplasty, autografts, or synthetic scaffolds is crucial as it helps establish their relative efficacy, safety, and outcome profiles. These comparisons are vital for guiding clinical decisions and optimizing treatment protocols for different tendon injuries [31,32]. Nonetheless, the theoretical and documented benefits—strong fixation, biological incorporation, and available length—make the Achilles bone–tendon allograft an attractive solution, especially in older or obese patients who may exhibit compromised tissue quality. Therefore, the primary goal of this research is twofold: first, to document the clinical, radiographic, and MRI findings in these two challenging cases and, second, to illustrate the broader viability of bone–tendon allografts in the published literature.

## 2. Materials and Methods

### 2.1. Study Design and Ethical Considerations

This observational analysis was conducted at the Orthopedic and Traumatology Clinic of “Victor Popescu” Military Emergency Hospital in Timișoara. Two patients with chronic, iterative tendon ruptures—one quadriceps tendon rupture and one Achilles tendon rupture—were included. Both patients had unsuccessful primary repairs that resulted in substantial tendon retraction and tissue deficits observed during follow-up periods through clinical assessments and imaging studies. The institutional review board approved this study protocol, and both patients provided informed consent for surgical procedures, imaging evaluations, and use of their anonymized clinical data for research and teaching. The study protocol was reviewed and approved by the institutional ethics committee, with approval reference number 20207/28, on 28 August 2024.

All procedures adhered to the ethical guidelines outlined in the Declaration of Helsinki. The specific inclusion criteria were as follows: (1) patients aged 18 years or older; (2) chronic tendon ruptures that have been present for at least six weeks, or identified at least four weeks following a missed diagnosis; (3) substantial tissue loss or retraction that makes direct tendon repair unfeasible; (4) previously active individuals aiming to return to moderate or high levels of physical activity post-surgery; (5) stable comorbidities that do not interfere with surgical recovery or rehabilitation; and (6) agreement to adhere to a structured postoperative rehabilitation protocol. The exclusion criteria were detailed as follows: (1) acute tendon ruptures occurring less than six weeks prior to the evaluation; (2) prior unsuccessful reconstructive surgery at the same tendon site; (3) active local or systemic infections; (4) medical conditions that compromise wound healing, such as uncontrolled diabetes, peripheral vascular disease, or chronic steroid use; and (5) patients unable to commit to follow-up schedules critical for postoperative assessment and data collection.

### 2.2. Surgical Technique

**Figure 1:**
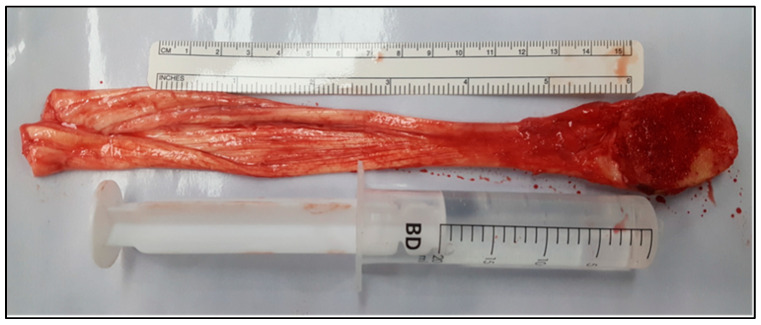
Aspect of allograft (quadriceps tendon).

**Figure 2:**
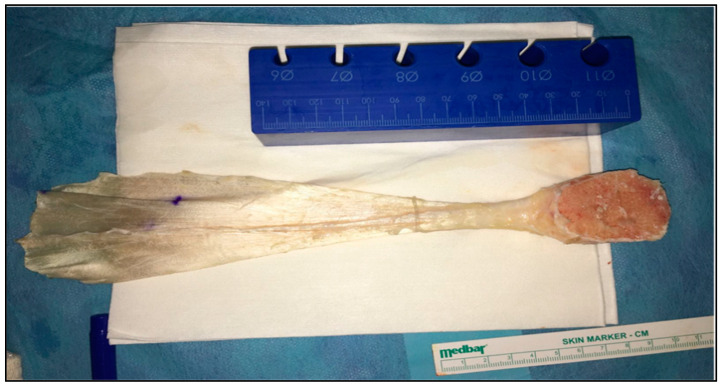
Aspect of allograft (Achilles tendon).

In the first case, an Achilles tendon bone–tendon allograft with a preserved calcaneal block was used to reconstruct a chronically ruptured quadriceps tendon (Figure 1 and Figure 2). The patella underwent an “L”-shaped osteotomy at its proximal pole to accommodate the calcaneal bone fragment. After securing the bone block with four compacting screws (Zimmer Biomet, Warsaw, IN, USA), the tendinous portion of the allograft was sutured to the remnant quadriceps mechanism using high-strength suture materials (Fiberwire, Arthrex, Naples, FL, USA) and a layered imbrication technique.

**Figure 3:**
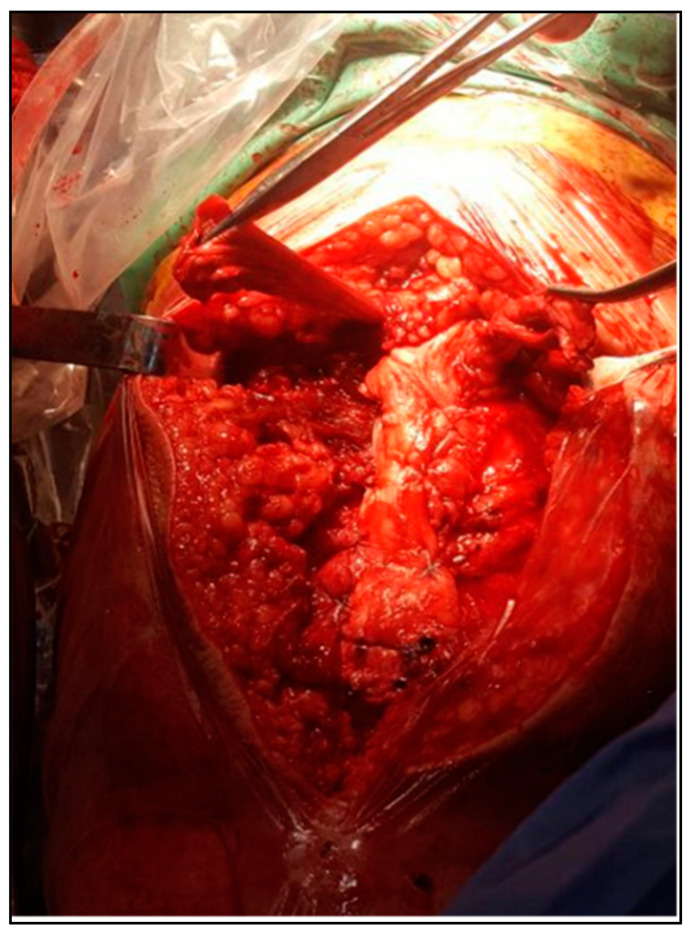
Final imbrication of quadriceps tendon.

**Figure 4:**
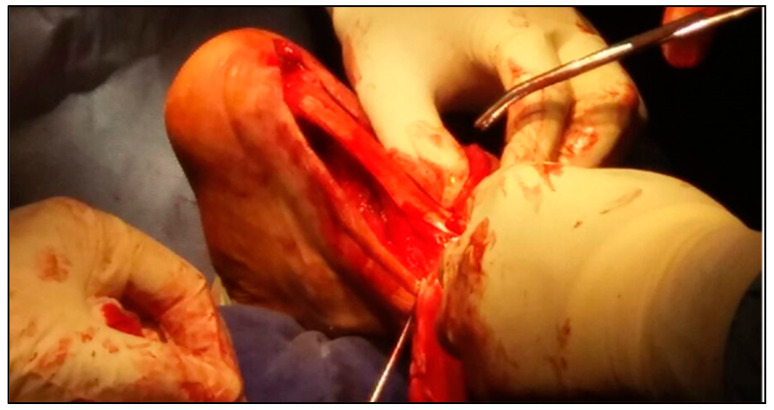
Final suture of Achilles tendon.

In the second case, the same type of allograft was utilized to reconstruct a large Achilles tendon defect. A “V”-shaped osteotomy was fashioned at the calcaneal tuberosity to receive the bone block from the allograft, which was then fixed with a bioabsorbable interference screw. The graft’s tendinous segment was split proximally and sutured to the remaining Achilles tendon in a layered fashion. Both procedures aimed to restore native tendon tension while maintaining stable bony fixation, as presented in Figure 3 and Figure 4.

### 2.3. Postoperative Protocol and Rehabilitation

**Figure 5:**
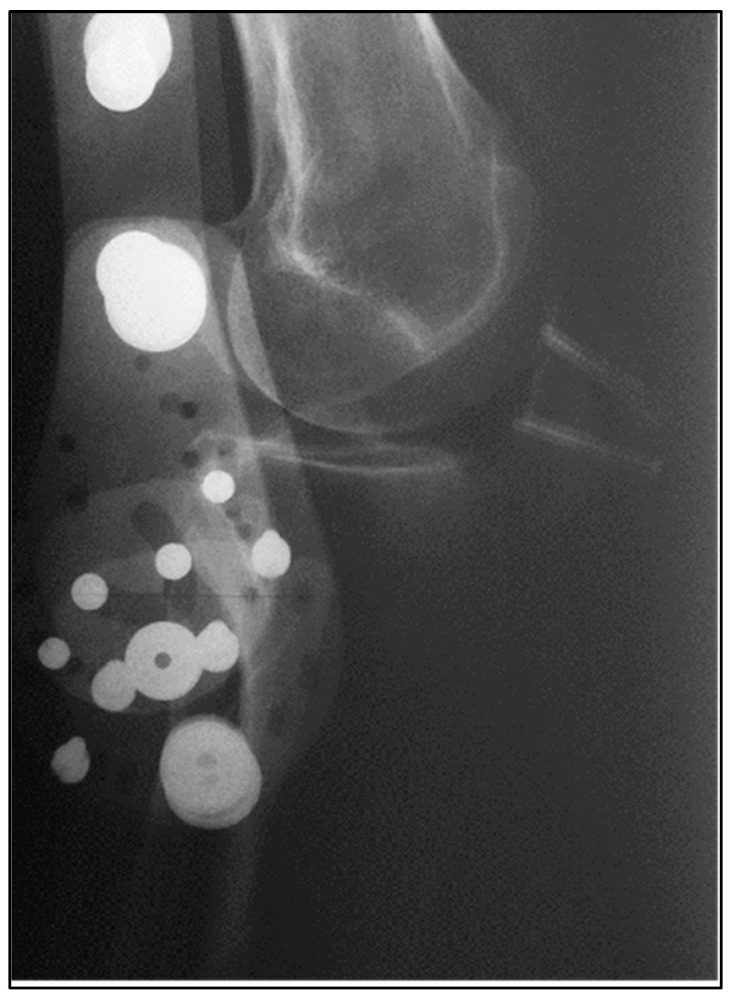
Postoperative radiograph—lateral knee incidence.

**Figure 6:**
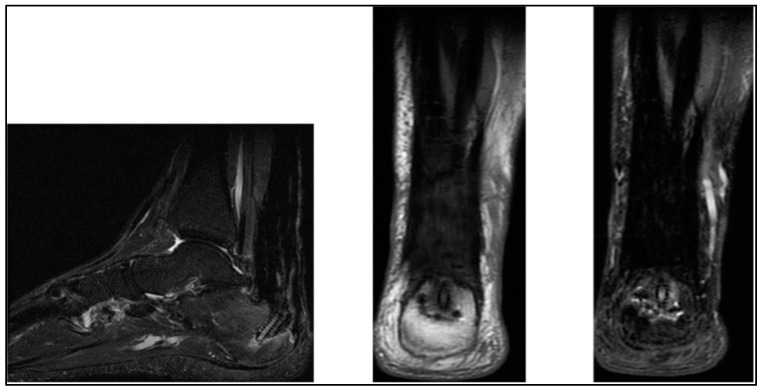
MRI view of Achilles tendon reconstruction.

Postoperatively, the quadriceps reconstruction patient was immobilized in a reinforced knee brace set in extension for six weeks, followed by a gradual increase in knee flexion range. Supervised physiotherapy focused on isometric quadriceps exercises, progressing to active resistance exercises by the eighth postoperative week. Follow-up evaluations were scheduled at 2, 6, 8, 12, and 24 weeks, and then at 6-month intervals. The Achilles tendon repair patient was immobilized in a removable ankle orthosis for six weeks. The ankle was maintained in plantar flexion for three weeks, and then neutral at 90° for another three weeks. Subsequent physiotherapy targeted regaining calf muscle strength (gastrocnemius and soleus), progressive weight-bearing, and functional gait retraining. Clinical follow-up and MRI assessments were performed using the same schedule as in the quadriceps patient (Figure 5 and Figure 6).

### 2.4. MRI Acquisition and Analysis

A 1.5-Tesla MRI system was employed to assess graft integrity at 8, 12, and 24 weeks postoperatively. The standard protocol included T1-, T2-, GRE’s, Proton Density (PD), STIR, and fat-suppressed sequences in multiple planes (sagittal, coronal, and axial). Special 3D Gradient Echo sequences were also acquired to visualize bone block healing and rule out edema or hardware migration. MRI findings were reviewed by two radiologists. Assessments centered on graft signal intensity, parallelism of tendon fibers, the presence of bone edema or cystic changes, hardware integrity, and evidence of fluid collections or re-rupture. Discrepancies in interpretation were resolved by consensus. Any concerning findings were correlated with clinical symptoms before determining further management steps.

### 2.5. Literature Review

A comprehensive electronic search was conducted in the PubMed, Web of Science, Embase, and Cochrane Library databases through December 2024. Search terms included combinations of “chronic tendon rupture”, “Achilles tendon”, “patellar tendon”, “posterior cruciate ligament”, “allograft”, “neglected rupture”, and “reconstruction”. Boolean operators (AND, OR) were used to maximize relevant article retrieval. A hand-search of article references and citations was also performed to identify additional relevant publications.

The initial screen included titles and abstracts, with two independent reviewers flagging potentially pertinent articles. Only full-text studies published in English were retained, focusing on adult patients (≥18 years) who underwent surgical reconstruction for chronic tendon ruptures (≥6 weeks from injury) involving the knee extensor mechanism or Achilles tendon, using allograft tissue for bridging. The following were excluded: studies of acute ruptures; animal or biomechanical-only investigations without clinical data; studies describing synthetic grafts or purely autograft reconstructions without an allograft cohort; and expert opinion pieces without patient-reported outcomes or detailed results.

Inclusion Criteria: (1) chronic rupture definition: rupture confirmed ≥6 weeks from initial injury or at least 4 weeks after a missed diagnosis for Achilles tendon injuries, or patellar tendon/PCL disruptions documented as iterative or neglected in the study text. (2) Patients underwent surgical reconstruction using any type of tendon allograft (Achilles, quadriceps, peroneus brevis, semitendinosus, etc.). (3) Articles providing at least one objective or subjective outcome measure at a minimum 6-month follow-up—preferably validated scoring (e.g., IKDC, Lysholm, Tegner, AOFAS, or ATRS). (4) Level I–IV evidence preferred, though due to limited availability, relevant case series (Level IV) or expert opinions (Level V) providing original patient data were also included.

Exclusion Criteria: (1) Studies of acute ruptures (<6 weeks post-injury). (2) Entirely autograft-based or synthetic-based reconstructions without an allograft arm. (3) Pediatric cohorts (patients < 18 years). (4) Purely biomechanical in vitro or animal studies without human clinical results. (5) The inability to extract allograft-specific data if combined with other methods.

The following categories were captured: (1) study characteristics: authors, the publication year, the study design (case series, retrospective, prospective, or systematic review), the sample size, and the level of evidence. (2) Patient demographics: mean age, sex distribution, time from injury to surgery, and comorbidities. (3) Surgical details: the tendon allograft type (Achilles, quadriceps, peroneus brevis, or bone-block presence), the fixation method (interference screws, transosseous sutures, or anchors), concomitant procedures (FHL transfer, xenograft augmentation, etc.). (4) Postoperative rehabilitation: weight-bearing timeline, immobilization duration, and range-of-motion protocols. (5) Outcomes: range of motion, validated scores (IKDC, Lysholm, Tegner, AOFAS, ATRS, FAOI, etc.), single-limb heel rise or quadriceps strength testing, radiographic measures (Telos Medical GmbH, Amöneburg, Germany), and KT-2000 (MEDmetric Corporation, San Diego, CA, USA), complication rates, and follow-up duration.

## 3. Results

Table 1 summarizes the fundamental preoperative profiles of the two cases. In Case 1, the patient was a 63-year-old male with morbid obesity (a BMI of 43.9), which is a notable risk factor for musculoskeletal stress and possible delayed tissue healing. His chronic quadriceps tendon rupture had been neglected after the failure of an initial reinsertion attempt. By the time he presented for allograft reconstruction, a sizeable 4 cm tendon gap was confirmed on MRI. This large defect, combined with his comorbidities, highlighted the complexity of his condition. In contrast, Case 2 involved a 42-year-old male with a history of a sports-related Achilles tendon rupture that was also iteratively torn following an insufficient primary repair. Despite being younger and otherwise healthy, his defect measured 7 cm, significantly larger than that of Case 1.

Table 2 presents the early postoperative outcomes at 8 weeks. The range of motion (ROM) was assessed during knee flexion for Case 1 and ankle flexion (plantar- and dorsiflexion combined) for Case 2. Although direct numerical comparisons are challenging because of the different joints, both patients demonstrated substantial progress from the immediate postoperative period. Case 1 achieved 0–110° of knee flexion, indicating a functional arc that allows ambulation with minimal discomfort. Case 2’s ankle ROM was recorded as 15–40° of flexion, which might seem limited but is still encouraging given the significant defect and the lengthy immobilization period required to protect the allograft.

Pain levels, evaluated via the Visual Analog Scale (VAS), were relatively low in both patients (3 for Case 1 and 2 for Case 2). This suggests that, although both individuals were still in the early healing phase, the reconstructive procedure and subsequent rehabilitation regimen did not lead to debilitating pain. One noticeable finding was the persistent muscle atrophy in both limbs—a 25% reduction in quadriceps circumference in Case 1 and a 30% reduction in calf circumference in Case 2. Such atrophy is common after prolonged immobilization and reduced weight-bearing and underscores the importance of targeted physiotherapy focused on muscle strengthening.

Table 3 outlines the MRI findings at 8 weeks, capturing the initial phase of graft healing and integration. Both reconstructions—quadriceps (Case 1) and Achilles (Case 2)—showed mildly increased T2 signals within the tendinous portion of the allograft. This finding is consistent with the normal postoperative inflammatory process, where local edema and increased vascularity contribute to a brighter T2 signal. The continuity of the graft fibers in both cases underscores the technical success of the reconstruction, as no areas of complete disruption or fluid gaps were seen on any imaging plane. Bone block integration is described as “early bridging” at the sites of osteotomy—“L”-shaped for the patella in Case 1 and “V”-shaped for the calcaneus in Case 2. Although the bone–tendon allograft approach provides robust fixation, complete osseous union at these interfaces takes several weeks to months to achieve. Neither patient exhibited MRI indicators of major complications, such as re-rupture, infection, or significant effusion.

Table 4 presents the mid-term (12-week) MRI status of both patients. A noteworthy improvement in graft signal intensity is observed on T1- and PD-fat-suppressed sequences, indicating progressive remodeling and diminished edema. The near-normal signal on T1 suggests that the collagen fibers within the graft organized more in line with native tendon architecture, supported by the “well-aligned” fiber orientation described. Bone block incorporation moved from early bridging toward partial bridging with stable positioning—an essential milestone for load-bearing. Such findings mirror the improved functional parameters, including a better ROM and increases in muscle strength. Notably, minimal scar tissue formation was seen in both cases, reflecting favorable healing rather than excessive fibroplasia or adhesions that could limit tendon glide or cause pain.

Residual pain was minimal (VAS of 1) in both individuals, suggesting that the risk of chronic pain or persistent inflammation is low. MRI scans confirmed stable, normal T2 signals within the allograft, indicative of well-organized collagen and the absence of edema or re-injury. Plain radiographs and cross-sectional imaging demonstrated complete bone block union without hardware complications—critical evidence supporting the effectiveness of using a calcaneal bone fragment in these reconstructions. Both patients were able to resume their respective occupations.

## 4. Review of Literature

Table 5 details the baseline characteristics of six studies evaluated in this review [10,33,34,35,36], illustrating a range of study designs, patient populations, and anatomical focuses. The oldest references revolve around neglected Achilles tendon ruptures (Ofili et al. [33], Deese et al. [34]) or patellar tendon reconstructions in TKA patients (Balato et al. [35]), while the more recent ones (Kim et al. [36], Kang et al. [37], Song et al. [10]) take the form of systematic reviews or retrospective comparisons, thus encapsulating a broader sweep of clinical experiences. Notably, sample sizes vary widely: from as few as 5 to 14 patients in specialized case series (Ofili et al. [33], Deese et al. [34]) to large meta-analyses or systematic reviews pooling anywhere from 35 to 238 patients (Song et al. [10], Balato et al. [35]).

The average patient age typically hovers around middle age (30 s–60 s), though certain series (Ofili et al. [33]) include older individuals, up to 77, presumably reflecting a demographic more prone to neglected rupture due to comorbidities or reduced awareness. The ratio of male to female participants is often unreported in detail, though many cohorts note a predominance of males—possibly linked to higher incidence of sports-related injuries. Follow-up intervals generally range from 12 to 27 months for the smaller series but can extend beyond 5 years for the larger systematic reviews (Kim et al. [36], Balato et al. [35]), though the latter aggregated heterogeneous follow-up data.

Anatomically, half of these studies center on chronic Achilles tendon ruptures (Song et al. [10], Ofili et al. [33], Deese et al. [34]), employing Achilles tendon allografts or alternative tendons (peroneus brevis) to reconstruct large tissue deficits. Kang et al. pivot to the knee, comparing Achilles vs. quadriceps tendon allografts in arthroscopic PCL reconstructions over roughly two years of follow-up. Meanwhile, Kim et al. [36] and Balato et al. [35] investigate extensor mechanism injuries, focusing on chronic patellar tendon disruptions—sometimes in post-TKA contexts—where surgeons have used either Achilles tendon bone–tendon allografts or complete extensor mechanism allografts to restore knee extension.

Table 6 focuses on the surgical approaches and the specific allograft constructs utilized in each study. Across the six studies, the Achilles tendon allograft emerges as the most commonly reported tissue, especially in contexts of chronic Achilles or patellar tendon ruptures. Several investigations (Deese et al. [34], Ofili et al. [33]) specifically mention using a calcaneal bone block attached to the Achilles tendon to facilitate bone-to-bone healing in the tibia, calcaneus, or patella. Meanwhile, Kim et al. [36] and Balato et al. [35] highlight that some reconstructive procedures for chronic patellar tendon ruptures used full extensor mechanism allografts (including patella–patellar tendon–tibial tubercle), while others used just the Achilles tendon bone–tendon segment.

Fixation methods vary but share common themes: interference screws (bioabsorbable or metallic) are frequently employed to secure the bone block within a precisely reamed tunnel. For purely tendinous segments (e.g., if the bone block is absent or was trimmed off), transosseous sutures or anchors are used to attach the graft to the host bone. In arthroscopic PCL reconstructions (Kang et al. [37]), both the femoral and tibial tunnels are stabilized with interference screws, supplemented by post-tie reinforcement or washers to mitigate potential graft slippage. Notably, some authors mention supplementary cerclage wires or xenograft overlays for added tensile support in large defect bridging (Kim et al. [36], Song et al. [10]).

Different rehabilitation protocols can significantly impact outcomes by influencing the rate of tissue healing, the risk of complications like re-rupture, and the restoration of function, with specific protocols tailored to the type of surgery and the anatomical site involved. Extensor mechanism repairs often necessitate locked extension braces for four to six weeks, followed by gradual range-of-motion exercises. In chronic Achilles reconstructions, practitioners commonly immobilize the ankle in equinus for about four to eight weeks before allowing progressive weight-bearing. Song et al. [10] note that certain teams permitted an earlier range of motion to reduce joint stiffness, but the risk of re-rupture or graft elongation remains a concern. Kang et al. [37] specify a PCL-specific protocol: non-weight-bearing for the first 4–6 weeks, partial weight-bearing thereafter, and progressive strengthening around the 10–12-week mark.

All studies documented significant postoperative functional improvements, whether measured by validated scoring instruments like Lysholm, IKDC, AOFAS, or by more subjective endpoints such as return to activity or single-limb heel raise. Kim et al. [36] and Balato et al. [35] highlight that patients with chronic patellar tendon ruptures do achieve robust gains in extension and relatively high knee scores, though certain subpopulations (especially older TKA patients) may experience residual deficits or extensor lag.

For PCL reconstructions, Kang et al. [37] confirm that both Achilles tendon allografts and quadriceps tendon allografts lead to better posterior knee stability (as evidenced by KT-2000 side-to-side differences decreasing from 8+ mm to around 3–4 mm) and improved Lysholm and Tegner activity scores. Interestingly, no statistical difference was detected between the two allograft types, indicating that surgeon preference or graft availability might guide the final selection.

In Achilles tendon-specific series (Song et al. [10], Ofili et al. [33], Deese et al. [34]), functional recovery timelines varied. Ofili et al. [33] reported that patients achieved single-limb heel rise by an average of 27 weeks. Deese et al. [34] found that four of five patients had minimal pain and resumed daily activities without re-rupture, though no standardized validated scale was used. Song et al. [10], a systematic review, underscores the paucity of validated measures—only a handful reported ATRS or AOFAS—and highlights consistently favorable outcomes with minimal complications (Table 7).

## 5. Discussion

The encouraging outcomes in both patients underscore the fundamental strengths of Achilles tendon bone–tendon allografts. By providing a robust bone block for secure fixation, these grafts minimize the risk of pullout in the early healing phase. Additionally, the tendinous component offers the length and tensile properties needed to bridge significant tissue gaps in chronic tendon ruptures.

The observed MRI signal changes in the study, such as mildly increased T2 signals and the progressive normalization of T1 signals, clinically indicate a successful initial integration of the grafts, with early vascular ingrowth and a reduction in inflammation. Over time, the normalization of MRI signals, along with the continuity of graft fibers and the progression of bone block integration, suggest the effective biological incorporation of the grafts, aligning with functional improvements seen in patient mobility and strength without significant complications. These findings support the potential for these allografts to restore tendon integrity and function in cases of severe chronic ruptures.

From a clinical standpoint, progressive strengthening and tailored physiotherapy protocols complemented the biological integration of the grafts. Although muscle atrophy was evident in both patients at 8 weeks, consistent rehabilitation allowed notable improvement by 12 and 24 weeks, demonstrating that the synergy of surgical reconstruction and incremental loading fosters robust tendon–bone healing.

Halvorson et al. [38] described an extensor mechanism reconstruction using an Achilles tendon allograft with suture tape augmentation for a chronic patella fracture, demonstrating that patellar height improved from a Caton–Deschamps index of 2.35 preoperatively to 1.11 at the 13-month follow-up. Their technique incorporated vertical screws in the patella for passing tape augmentation sutures and reported a functional arc of motion reaching 90° of knee flexion. In a similar manner, Mercer et al. [39] used a combination of semitendinosus autograft and Achilles allograft to treat a chronic rectus femoris myotendinous junction rupture. They found that careful tensioning and fixation allowed for secure graft incorporation and reduced mechanical stress, ultimately leading to a significant improvement in the knee range of motion and the restoration of rectus femoris function. Both reports are relevant to the current investigation in that they emphasize the unique capacity of Achilles allografts to bridge substantial gaps and to achieve firm anchorage, supporting the goal of managing iterative, chronically ruptured tendons (whether quadriceps or Achilles) in patients who might otherwise have limited local tissue stock.

Regennass et al. [40] evaluated Achilles tendon allograft with ipsilateral semitendinosus reinforcement for chronic patellar tendon ruptures in two patients and noted early gains in their range of motion, with one patient achieving up to 80° of knee flexion by 3 months and improved walking capacity thereafter. The authors observed an uncomplicated incorporation of the semitendinosus frame into the Achilles graft, contributing to stable load distribution and accelerated recovery. In a similar manner, Pontoh et al. [41] reported using ipsilateral semitendinosus tendon autograft to repair a chronic quadriceps tendon rupture. Their patient regained a 0° to 130° knee flexion arc by the 6-month mark, highlighting that autograft harvest can be advantageous in younger individuals who are able to sustain a more aggressive rehabilitation protocol without substantial donor site morbidity. These findings lend practical support to this study, which seeks to extend the viability of bone–tendon allografts to instances where direct repair or lesser grafts prove inadequate, particularly if the tendon gap exceeds several centimeters or is compromised by poor tissue integrity.

Maier et al. [42] described a revision quadriceps tendon re-rupture reconstruction with an Achilles allograft, emphasizing the difficulty of maintaining vascular integrity and combating scar tissue in re-injured tissue. They integrated a pseudo-Pulvertaft weave within the native quadriceps tendon and anchored the graft distally through patellar bone tunnels, achieving secure fixation and promoting biologic incorporation around the graft–tendon interface. In a similar manner, Permutt et al. [10] presented a case of chronic quadriceps rupture in which Achilles allograft was paired with a V-Y tendon plasty and augmented by poly-tape. They noted successful early return to daily activities and substantial structural integrity of the augmented construct despite poor native tissue quality and a large defect measuring several centimeters. These two studies resonate with the current approach, as this research highlights not only the biomechanical strength and biologic integration of Achilles tendon allografts but also the additional stability conferred by bone-block fixation in older or obese patients who often present with complex, chronic tears.

Miller et al. [43] used dermal allograft augmentation in a chronic quadriceps tendon rupture where extensive tissue degeneration was observed. Their surgical technique—employing anchors and a “broad compression” across the tendon–bone interface—was predicated on published re-rupture rates as high as 10% in chronic injuries. They observed improved structural integrity of the tendon by using the dermal graft for additional tensile support. In a similar manner, Okay et al. [44] presented a reconstruction of a previously failed quadriceps tendon repair using an Achilles allograft secured with suture anchors. At the 6-year follow-up, the patient demonstrated 135° of flexion and near-complete restoration of extensor strength, indicating that robust fixation to the superior patella and double-layered graft suturing promoted lasting joint stability. These investigations bolster the rationale behind the current study, which shows that adding an Achilles bone–tendon allograft can be decisive in restoring tendon continuity while providing a biologic scaffold for healing, thus responding directly to the recognized lack of robust comparative trials favoring any one reconstructive method.

Dandu et al. [45] discussed a revision strategy for chronic quadriceps tendon repair by augmenting with an Achilles allograft bone plug, which was affixed to the tibia via an interference screw to foster bone-to-bone healing and was then sutured proximally in a tensioned Krackow manner. The authors referenced prior studies of 17 similar reconstructions showing durable long-term outcomes, with extensor lags of less than 3° on average and improved quadriceps strength. In a similar manner, Hoang et al. [46] used an Achilles tendon bone block allograft to manage a chronic 3-week-old quadriceps rupture, emphasizing that the bone block was shaped to 1 × 1 cm and placed at the distal patella. They reported satisfactory tension in the extensor mechanism, tight bone-to-bone healing, and a restoration of pain-free motion arcs, concluding that this approach offered a strong construct for large tendon gaps or severely diminished tissue quality.

This aligns closely with the current research objectives, in which we illustrate that securing the bone block within an osteotomy site provides a stable framework for allograft incorporation, enabling rapid functional progress. By adding detailed clinical, radiographic, and MRI data for two such challenging cases, this work offers further evidence that Achilles bone–tendon constructs may bridge extensive tendon defects effectively—precisely addressing the literature gap regarding comparative efficacy versus V–Y plasty, autograft, or synthetic mesh alternatives.

Nevertheless, the current study has several limitations that must be acknowledged. Firstly, the observational analysis included only two patients, limiting the generalizability of the findings. The absence of a control group and the small sample size hinder the ability to definitively conclude the superiority of Achilles allografts over traditional methods. Additionally, the study’s reliance on short-term follow-up data (up to 24 weeks) may not adequately capture long-term outcomes such as graft durability and the risk of late complications.

## 6. Conclusions

Achilles tendon bone–tendon allografts demonstrate significant efficacy in reconstructing chronic quadriceps and Achilles tendon ruptures, providing robust fixation and promoting biological integration. The positive clinical and radiographic outcomes observed in this study, alongside supportive findings from the literature, indicate that Achilles allografts offer a viable and potentially superior alternative to traditional repair strategies, particularly in cases involving large defects and compromised tissue quality. These results underscore the importance of advanced graft techniques in enhancing functional recovery and restoring tendon continuity in challenging chronic rupture scenarios. Future research should focus on larger comparative trials to further validate the advantages of allograft constructs and optimize surgical protocols for improved patient outcomes.

## Figures and Tables

**Table 1:** Characteristics of patients and inflammatory markers.

Inflammatory Markers	Variable	Case 1: Quadriceps Tendon Rupture	Case 2: Achilles Tendon Rupture
	Age (years)	63	42
	Sex	Male	Male
	BMI (kg/m^2^)	43.9	26.5
	Time Since Initial Rupture (weeks)	12	16
	Defect Length (cm)	~4	~7
	Previous Surgery (Failed Repair)	Yes	Yes
	Comorbidities	Obesity, Hypertension	None
C-Reactive Protein (mg/L)	Baseline	10	8
	8 weeks	6.8	5.4
	12 weeks	3.5	2.9
	24 weeks	1.7	1.4
WBC (×10^9^/L)	Baseline	7.5	6.8
	8 weeks	6.2	5.5
	12 weeks	5.1	4.9
	24 weeks	4.6	4.3
TNF-alpha (pg/mL)	Baseline	74	68
	8 weeks	58	53
	12 weeks	42	39
	24 weeks	30	27

BMI—body mass index; WBC—white blood cells; TNF—tumor necrosis factor.

**Table 2:** Postoperative clinical assessment at 8 weeks.

Parameter	Case 1 (Quadriceps)	Case 2 (Achilles)
Knee/Ankle ROM (degrees)	0–110° knee flexion	15–40° ankle flexion
Pain (VAS 0–10)	3	2
Quadriceps/Calf Circumference†	−25% vs. contralateral	−30% vs. contralateral
Weight-Bearing Status	Partial	Partial

**Table 3:** MRI evaluation at 8 weeks.

MRI Parameter	Case 1 (Quadriceps)	Case 2 (Achilles)
Graft Signal Intensity	Mildly increased on T2	Mildly increased on T2
Fiber Continuity	Continuous alignment, minor edema	Continuous alignment, minor edema
Bone Block Integration	Early bridging at the patella	Early bridging at the calcaneus
Hardware Artifact	Minimal, no screw loosening	Minimal, no screw loosening
Signs of Complication	None	None

**Table 4:** MRI evaluation at 12 weeks and 24 weeks.

MRI Parameter	Case 1 (Quadriceps)	Case 2 (Achilles)
12 Weeks Evaluation		
Graft Signal Intensity	Near-normal on T1, mild on PD-FS	Near-normal on T1, mild on PD-FS
Fiber Orientation	Well aligned, reduced edema	Well aligned, reduced edema
Bone Block Incorporation	Partial bridging, stable positioning	Partial bridging, stable positioning
Soft Tissue Reaction	Minimal scar tissue seen	Minimal scar tissue seen
24 Weeks Evaluation		
Residual Pain (VAS 0–10)	1	1
Graft Integrity (MRI)	Normal T2 signal, intact fibers	Normal T2 signal, intact fibers
Bone Block Union (X-ray)	Complete union, no hardware issues	Complete union, no hardware issues
Return to Work/Sports	Yes, resumed normal duties	Yes, resumed normal duties

**Table 5:** Baseline characteristics of included studies.

Study/Year	Design and Level of Evidence	Sample Size	Mean Age (Years)	Chronic Rupture Site	Follow-Up Range (Months)
Kim et al. (2022) [36]	Systematic review	96 patients (pooled)	30–62	Chronic patellar tendon injuries, multiple grafts	21–68
Kang et al. (2019) [37]	Retrospective cohort	29	17–53 (Achilles group) 19–46 (Quad group)	PCL reconstructions: Achilles vs. quadriceps tendon allografts	24–26
Song et al. (2019) [10]	Systematic review	35 (across 9 studies)	20–60	Chronic Achilles tendon ruptures: allografts (Achilles, PB)	12–30
Ofili et al. (2016) [33]	Retrospective case series	14	34–77	Neglected Achilles tendon ruptures with Achilles allografts	12–27
Balato et al. (2023) [35]	Systematic review and meta-analysis	238 (pooled)	54–74	Extensor mechanism reconstructions after TKA, incl. ATA/EMA	12–60
Deese et al. (2015) [34]	Case series	5	32–59	Chronic Achilles ruptures, Achilles tendon allograft w/bone block	12

**Table 6:** Surgical techniques and graft details.

Study	Allograft Type	Fixation Method	Augmentation/Concomitant Procedure	Rehabilitation Protocol
Kim et al. (2022) [36]	Mixed: Achilles, hamstring, BTB, synthetic	Transosseous tunnels, screws, anchors (various)	Some with cerclage wires, hardware augmentation	Typically locked in extension for ~6 wks, progressive ROM
Kang et al. (2019) [37]	Achilles tendon allograft vs. quadriceps	Bioabsorbable interference screws (femoral/tibial)	None specifically; meniscal repairs if needed	PCL rehab protocol: NWB 4–6 wks, partial WB after 6 wks
Song et al. (2019) [10]	Primarily Achilles tendon; 1 peroneus brevis	Screws for bone block or suture anchor for tendon portion	Some used FHL transfers, xenograft overlays	Varied: some immediate ROM, some cast immobilization
Ofili et al. (2016) [33]	Achilles tendon allograft (14 patients)	Bone block in 2/14, fixated with screws + suture bridging	None specifically, except 1 MCL repair in data	6–8 wks cast or boot in equinus, then PT progression
Balato et al. (2023) [35]	ATA (Achilles tendon allograft) vs. EMA	Metal or bioabsorbable screws, sometimes wire augmentation	Some subgroups with entire extensor mechanism	Typically 4–6 wks locked extension, gradual PT
Deese et al. (2015) [34]	Achilles tendon w/calcaneal bone block (5)	Single screw fixation for bone block, suture anchor proximally	No additional tendon transfer reported	Gradual progression from NWB to FWB, ~8–10 wks

**Table 7:** Clinical outcomes and functional measures.

Study	Primary Outcome Scores	Functional Milestones	Notable Observations	Complications
Kim et al. (2022) [36]	Lysholm (70–94), IKDC subjective (median ~75–80), SF-36	Knee flex 0–120°, minimal extensor lag in majority; improved PROM	All reconstructive methods improved function, no single best method	Pain (common), extensor lag, and wire breakage in some cases. Graft failure <1% overall. Infection ~2%.
Kang et al. (2019) [37]	KT-2000 side-to-side difference, Telos x-ray, Lysholm, Tegner, IKDC subjective	Both Achilles and quadriceps groups showed significant improvement in posterior stability, Lysholm up by ~16–25 points	No difference in final functional scores between allograft types	No major infections. No re-ruptures. Delayed union not observed.
Song et al. (2019) [10]	AOFAS (90–100 for some), ATRS (80–100 in select cases), partial single-limb heel rise data	Return to normal activity around 12–24 weeks in small cohorts, minimal re-ruptures reported	Heterogeneity: only 1–2 studies used validated Achilles tendon measures (ATRS)	Delayed wound healing, infection, heterotopic ossification occasionally in Achilles reconstructions.
Ofili et al. (2016) [33]	Time to single-limb heel rise (mean 27 wks), normal shoe gear at ~13.5 wks, no validated PROM used	100% achieved unassisted single-heel raise, zero re-ruptures	Predominantly older demographic; Achilles allograft bridging ~7 cm defect	1 delayed union (7%) of bone block, no infection, no re-rupture.
Balato et al. (2023) [35]	KSS or other knee scores (70–85 range), extensor lag < 10° in many, some had re-rupture rates~23% in meta-analysis but not all due to allografts	Most patients > 100° flexion, improved ambulatory status, but persistent deficits in older TKA pop.	Achilles tendon allografts vs. extensor mechanism allografts had similar success	Overall failure 23% in large meta-analysis, but not all allograft.
Deese et al. (2015) [34]	No formal validated scoring system, reported return to daily activities, no chronic pain in 4/5 patients	Full weight-bearing ~10 wks, no re-rupture, 1 partial bone block fragmentation	Small sample, ~7.6 cm average gap bridged with Achilles allograft bone block	1 deep infection, 1 partial fragmentation of bone block, 1 delayed incision healing.

## Data Availability

The data presented in this study are available on request from the corresponding author.
